# Is aspiration an effective acute stroke treatment in older adults?

**DOI:** 10.3389/fneur.2023.1149531

**Published:** 2023-05-02

**Authors:** Jerzy Narloch, Adam Piasecki, Piotr Ziecina, Aleksander Dȩbiec, Marek Wierzbicki, Jacek Staszewski, Piotr Piasecki

**Affiliations:** ^1^Department of Interventional Radiology, Military Institute of Medicine–National Research Institute, Warsaw, Poland; ^2^Faculty of Medicine, Medical University of Warsaw, Warsaw, Poland; ^3^Department of Neurology, Military Institute of Medicine–National Research Institute, Warsaw, Poland

**Keywords:** stroke, aspiration, elderly, thrombectomy, interventional radiology

## Abstract

**Introduction:**

Clinical outcomes after interventional stroke treatment rely on several factors, with older age being associated with poorer results, which are mainly attributed to patient's comorbidities and medications. The delivery of an aspiration catheter could be hindered by carotid tortuosity, which is more prevalent in elderly patients with increasing age. In this study, we aimed to compare the clinical and angiographic outcomes of a direct aspiration first-pass technique in interventional stroke treatment for elderly patients compared with younger patients.

**Materials and methods:**

A total of 162 patients (92 women and 70 men, aged between 35 and 94 years +/– 12.4 years) were included in this study. Patients who were treated in a comprehensive stroke center due to a large-vessel occlusion stroke using aspiration as the first-choice treatment were included in this study. To evaluate carotid arteries, the tortuosity index (TI) was calculated for each segment of each carotid pathway.

**Results:**

Age correlated significantly with the presence of carotid tortuosity (*R* = 0.408, *p* = 0.000), extracranial length ratio (*R* = 0.487, *p* = 0.000), and overall length ratio (*R* = 0.467, *p* = 0.000). No significant associations were found with coiling, kinking, or intracranial length ratio. Successful aspiration-based recanalization rate decreased with increasing age, and the differences between the age subgroups were not statistically significant. A comparison of the extreme subgroups, i.e., <60 years old vs. ≥80 years old, did not yield a statistically significant change (*p* = 0.068).

**Conclusion:**

Successful aspiration-based recanalization rate decreased with increasing age; however, these differences were not significant. Clinical outcomes did not significantly differ with regard to carotid tortuosity, regardless of the time of assessment. Neither intracranial nor extracranial tortuosity was significantly associated with reperfusion-related complications in either of the age subgroups.

## Introduction

Clinical outcomes after interventional stroke treatment rely on several factors. Older age is associated with poorer results, which are mainly attributed to patient's comorbidities and medications. Successful recanalization after a single retrieval maneuver is the primary goal of treatment in cases of acute ischemic stroke, and it is an independent factor for good clinical outcomes, regardless of age (1–4).

The results of the ASTER and COMPASS trials have established that, compared with stent-retriever-based techniques, “a direct aspiration first-pass technique” (ADAPT) was a non-inferiority trial in terms of successful recanalization and functional outcomes. Moreover, ADAPT has gained an increasing number of proponents due to its lower cost and faster procedure time (5–7).

Carotid tortuosity, which becomes more prevalent with increasing age, can hinder the delivery of the aspiration catheter (8). Moreover, the navigation of aspiration catheters is further impeded by the branching of the parent artery (in particular, the ophthalmic branch of the internal carotid artery [ICA]). Unfavorable anatomy poses technical challenges that lead to increased procedure time and delays in reperfusion, which affect patient outcomes (5, 8–10).

In this study, we aimed to compare the clinical and angiographic outcomes of elderly patients who received ADAPT as part of interventional stroke treatment with the clinical and angiographic outcomes of younger patients.

## Materials and methods

### Study design

The primary goal of this study was to assess whether aspiration alone could successfully treat stroke that is caused by a large-vessel occlusion in elderly patients compared with younger patients. Data regarding the modified Rankin score at 1, 3, and 12 months; National Institute of Health Stroke Score (NIHSS) before and after stroke treatment; time from onset-to-groin access; time from onset-to-recanalization; successful reperfusion rate (mTICI 2b-3) (11); first-pass success rate (mTICI 2b-3); periprocedural complication rate; anatomical characteristics; the presence and grade of vessel tortuosity; and aspiration catheter parameters were collected and analyzed retrospectively. The patients were divided into the following groups according to their age: < 60 years, ≥60 years, < 65 years, ≥65 years, < 80 years, and ≥80 years. The groups were created in accordance with the following criteria based on the official definition of old age by the United Nations: 65 years is the most frequently reported age of the patients at the time of hospitalization for interventional stroke treatment, and octogenarians constituted the oldest age group in our study (12). Hemorrhagic transformation of an ischemic infarct was classified according to the European Cooperative Acute Stroke Study (ECASS II) (13).

### Study population

Consecutive patients who underwent ADAPT as the first choice of treatment for the treatment of stroke due to a large-vessel occlusion between January 2016 and August 2021 at a comprehensive stroke center were included in the present study. All patients were eligible to receive endovascular treatment according to the American Heart Association/American Stroke Association (AHA/ASA) and European Stroke Organization (ESO)—European Society of Minimally Invasive Neurological Therapy (ESMINT) guidelines. The eligible patients received 0.9 mg/kg of an intravenous recombinant tissue plasminogen activator (rtPA) (14).

### Endovascular treatment

All procedures were performed by experienced interventional radiologists (who had performed > 50 endovascular stroke treatment procedures) *via* groin access. Mechanical thrombectomy was performed under local or general anesthesia, depending on the Glasgow Coma Scale (GCS) status of the patient. According to the institutional guidelines, ADAPT was chosen as the first choice of treatment, provided the aspiration catheter was delivered to the occlusion site. After three unsuccessful attempts at aspiration, the stent-retriever was used to continue rescue thrombectomy and such cases were excluded from the analysis.

To confirm the cerebral large-vessel occlusion (LVO), an Impress diagnostic peripheral catheter (Merit Medical, South Jordan, UT, USA) was placed in an 8F Radifocus Super Arrow-Flex Sheath Introducer (Arrow International Inc., PA, USA). The diagnostic catheter was exchanged for a guiding catheter, such as Neuron MAX (Penumbra, Inc., Alameda, CA, USA) or Fubuki (Terumo-MicroVention, Tustin, CA, USA). The following aspiration catheters were used in this study: the React™ 68 and React™ 71 (Medtronic, Minneapolis, MN, USA); SOFIA and SOFIA Plus (Terumo-MicroVention, Tustin, CA, USA); AXS Catalyst 5, 6, and 7 (Stryker, Kalamazoo, MI, USA); or ACE 68, JET7 (Penumbra Inc., Alameda, CA, USA). A Microcatheter Headway 0.021” (Terumo-MicroVention, Tustin, CA, USA) with 0.014” microwire Traxcess (Terumo, Tokyo, Japan) was used to reach the occlusion site. Aspiration passes were performed using ADAPT as described previously (15).

### Imaging technique

Computed tomography angiography (CTA) was performed for the region extending from the aortic arch to the vertex of the skull using a 64-row multidetector CT scanner (General Electric LightSpeed VCT, GE Healthcare, Milwaukee, WI, USA) with a helical technique using the following parameters:120 kV, 400 mA, collimation: 40 × 0.625 mm, rotation: 0.5 s, and pitch factor: 0.984.

Automatic bolus triggering at the aortic arch was performed by administering a contrast medium (iomeprol, 350 mg I/mL, Iomeron, Bracco, Milan, Italy) *via* the antecubital vein with an 18-gauge cannula using a coupled power injector (a flow rate of 5 mL/s, 70 mL of contrast medium was injected followed by a 50-mL saline flush).

### Image processing and analysis

Dedicated software for visualizing vessel tortuosity was used for the analysis. VesselIQ Xpress (GE Healthcare, Milwaukee, WI, USA) is a post-processing application for the Advantage Workstation (AW) platform that is connected to a local PACS that can be used to analyze two- and three-dimensional CTA images and the data derived from DICOM 3.0 compliant CT scans. It extracts bony structures for the accurate identification of the vessels. The vessels were evaluated in a curved reformat, lumen, or multiple-plane reformat (MPR) view. Tools available for sizing the vessel were utilized once the vessels were visualized.

**Figure 1:**
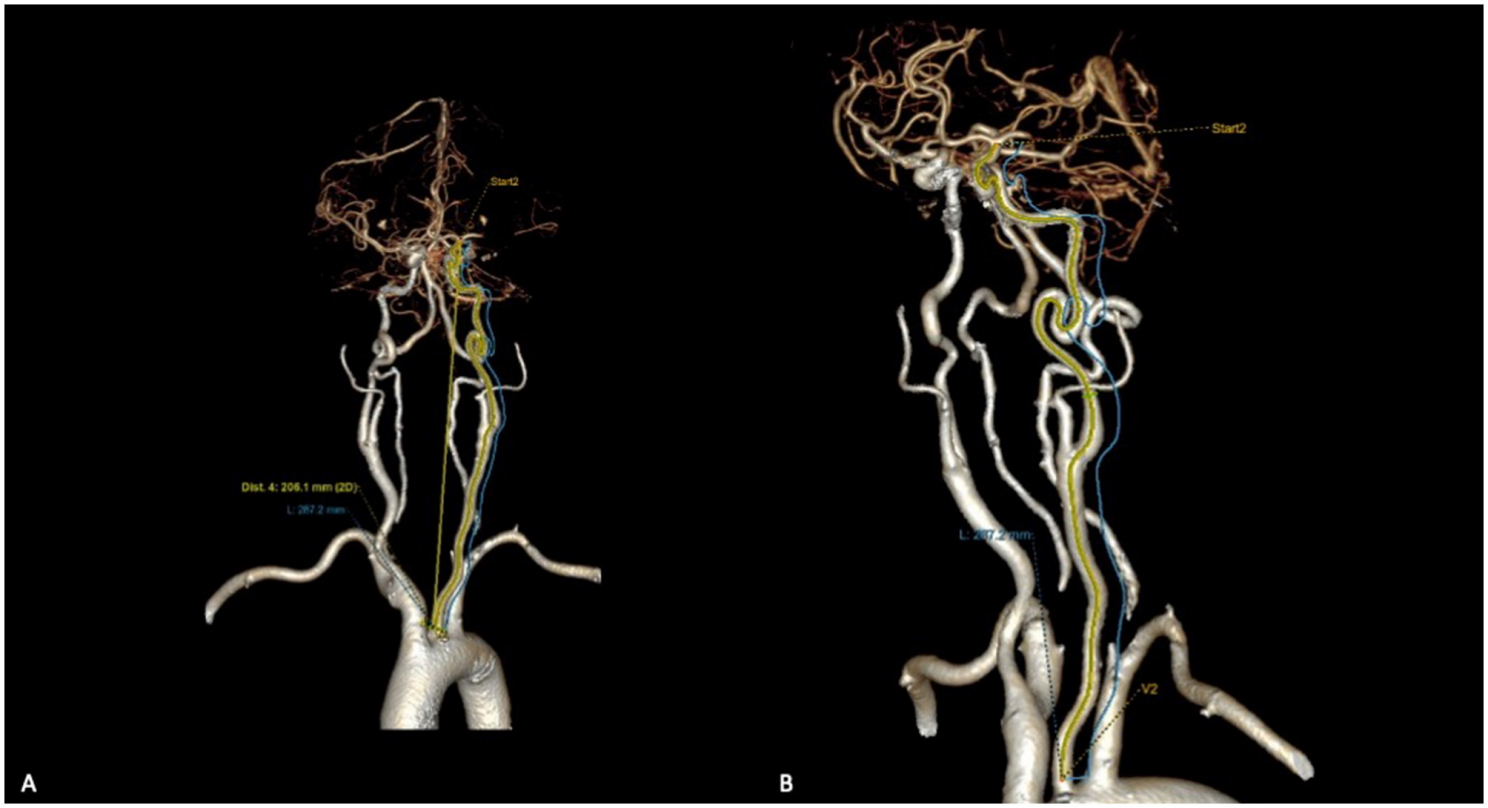
Performance of ADAPT in selected age groups. However, none of the differences were statistically significant.

The carotid artery pathway along the centerline was plotted in a three-dimensional space and divided into two main segments: (1) from the aortic arch takeoff (brachiocephalic artery [BCA]/common carotid artery [CCA]) of CCA to the entry of ICA into the skull and (2) intracranial ICA to the bifurcation into the middle cerebral artery (MCA) and anterior cerebral artery (ACA). The tortuosity index (TI) was calculated for each segment of each carotid pathway using the following formula: extracranial TI + intracranial TI = [(centerline distance)/(straight-line distance)−1] × 100. The straight length of the carotid artery pathway was measured individually for the overall length, extracranial, and intracranial segments in the anteroposterior position. Figure 1 presents a sample case of the measurement procedure.

The vertebral artery (VA) from the takeoff to the bifurcation of the basilar artery (BA) was measured in the case of posterior circulation, and two separate segments were created: extracranial and intracranial.

The carotid artery and VA selected for MT access were evaluated. In cases with the occlusion present proximal to the distal measurement landmarks, the contralateral side was evaluated as a surrogate. Using the previously established modified criteria by Wiebel–Fields and Metz (8, 16, 17), dolichoarteriopathy was visually identified and defined as tortuosity, kinking, and coiling.

The images were reviewed and evaluated by two experienced neuroradiologists.

### Statistical analysis

The recorded data included categorical variables such as: the modified Rankin score at 1, 3, and 12 months, presence of vessel tortuosity, type of aortic arch, type of periprocedural complications (vasospasm, dissection, embolization in a new territory, subarachnoid hemorrhage, and intracranial hematoma), presence of successful reperfusion (mTICI 2b-3), and presence of first-pass success.

Continuous data included the National Institute of Health Stroke Score (NIHSS) before and after stroke treatment; time from onset-to-groin access; time from onset-to-recanalization; aspiration catheter parameters (internal diameter and external diameter), occluded vessel diameter, and length ratios.

The patients were divided into the following groups according to their ages: < 60 years, ≥60 years, < 65 years, ≥65 years, < 80 years, and ≥80 years. The aforementioned data were compared between these different age groups.

Student's *t*-test and Mann–Whitney U test were used for estimating continuous data. The chi-squared or Fisher's exact test was used for estimating categorical variables. The 95% confidence interval (CI) was calculated. Statistical significance was set at a value of *P* of < 0.05.

## Results

In total, 162 patients met the inclusion criteria in this study. Overall, 92 women and 70 men, aged between 35 and 94 years (+/– 12.4 years), were included in this study. The baseline characteristics, clinical and radiological data of treated patients stratified according to the subgroup are summarized in Table 1.

**Table 1:** Baseline characteristics, clinical and radiological data of treated patients, divided into age groups: < 60 years vs. ≥60 years, < 65 years vs. ≥65 years, and < 80 years vs. ≥80 years.

**Age group**	** < 60**	**≥60**	***p*-value**	** < 65**	**≥65**	***p*-value**	** < 80**	**≥80**	***p*-value**
	**N/mean**	**N/mean**		**N/mean**	**N/mean**		**N/mean**	**N/mean**	
Sex	**30 pts**	**132 pts**		**53 pts**	**109 pts**		**128 pts**	**34 pts**	
*Women/MEN*	12 (40%)/18 (60%)	80 (61%)/52 (39%)	0.032	21 (40%)/32 (60%)	71 (65%)/38 (35%)	0.002	63 (49%)/65 (51%)	29 (85%)/5 (15%)	0.000
Pre-stroke MRS			0.110			0.032			0.058
***0***	28	88		43	73		98	18	
***1***	0	18		1	17		10	8	
***2***	0	3		0	3		2	1	
***3***	0	1		0	1		1	0	
***4***	0	0		0	0		0	0	
***5***	0	3		0	3		2	1	
Side			0.719			0.306			0.567
***Left***	17	66		27	56		67	16	
***Right***	11	59		21	49		53	17	
***Posterior***	2	7		5	4		8	1	
Tandem *yes/no*	8 (27%)/22 (73%)	22 (17%)/109 (83%)	0.160	15 (28%)/38 (72%)	15 (14%)/93 (86%)	0.025	27 (21%)/100 (79%)	3 (9%)/31 (91%)	0.074
Occlusion			0.580			0.525			0.457
***ICA-T***	12	40		20	32		44	8	
***MCA-M1***	14	63		22	55		60	17	
***MCA-M2***	2	21		6	17		16	7	
***BA***	2	6		4	4		7	1	
***PCA-P2***	0	2		1	1		1	1	
rTPA *Yes/No*	23 (77%)/7 (23%)	85 (64%) /47 (36%)	0.141	40 (75%)/13 (25%)	68 (62%)/41 (38%)	0.068	87 (68%)/41 (32%)	21 (62%)/13 (38%)	0.313
Complications *Yes/No*	3 (10%)/27 (90%)	17 (13%) /115 (87%)	0.470	5 (9%)/48 (91%)	15 (14%)/94 (86%)	0.304	13 (10%) /115 (90%)	7 (20%)/27 (80%)	0.092
Hemorrhage 24 h *Yes/No*	7 (23%)/20 (67%)	34 (26%)/78 (59%)	0.524	15 (28%)/33 (72%)	27 (25%)/65 (75%)	0.885	31 (24%) /81 (63%)	11 (32%)/17 (50%)	0.336
mRS at discharge			0.073			0.156			0.083
***0***	4	7		5	6		10	1	
***1***	5	14		6	13		15	4	
***2***	4	17		9	12		19	2	
***3***	3	10		6	7		12	1	
***4***	1	11		2	10		8	4	
***5***	7	38		14	31		33	12	
***6***	5	30		9	26		26	9	
Discharged			0.316			0.224			0.044
***Home***	11	34		15	30		35	10	
***Rehab***	6	29		14	21		31	4	
***Care home***	1	14		2	13		8	7	
***Other***	3	4		4	3		6	1	
***Home** **+** **rehab***	3	10		6	7		12	1	
mRS at 30 days			0.145			0.330			0.046
***0***	3	8		4	7		9	2	
***1***	6	9		6	9		14	1	
***2***	3	16		7	12		17	2	
***3***	3	18		8	13		19	2	
***4***	0	12		2	10		8	4	
***5***	8	33		15	26		31	10	
***6***	5	24		7	22		21	8	
mRS at 90 days			0.088			0.127			0.011
***0***	1	5		1	5		4	2	
***1***	5	9		6	8		14	0	
***2***	5	17		10	12		19	3	
***3***	0	11		2	9		9	2	
***4***	2	12		5	9		13	1	
***5***	3	11		6	8		12	2	
***6***	1	18		2	17		10	9	
mRS at 12 months			0.013			0.038			0.002
***0***	1	1		1	1		2	0	
***1***	6	6		6	6		12	0	
***2***	0	10		5	5		9	1	
***3***	2	7		4	5		8	1	
***4***	0	4		1	3		3	1	
***5***	0	1		0	1		1	0	
***6***	1	15		3	13		9	7	
AF *Yes/No*	9 (30%)/21 (70%)	57 (43%)/75 (57%)	0.131	13 (25%)/40 (75%)	53 (49%)/56 (51%)	0.003	43 (34%)/85 (66%)	23 (68%)/11 (32%)	0.000
DB *Yes/No*	3 (10%)/27 (90%)	34 (26%)/98 (74%)	0.047	8 (15%)/45 (85%)	29 (27%)/80 (73%)	0.073	27 (21%)/101 (79%)	10 (29%)/24 (71%)	0.210
LIP *Yes/No*	12 (40%)/18 (60%)	57 (43%)/75 (57%)	0.457	19 (36%)/34 (64%)	50 (46%)/59 (54%)	0.149	55 (43%)/73 (57%)	14 (41%)/20 (59%)	0.505
NT *Yes/No*	12 (40%)/18 (60%)	93 (70%)/39 (30%)	0.002	24 (45%)/29 (55%)	81 (74%)/28 (26%)	0.000	80 (63%)/48 (37%)	25 (74%)/9 (26%)	0.160
COR *Yes/No*	4 (13%)/26 (87%)	33 (25%)/99 (75%)	0.126	10 (19%)/43 (81%)	27 (25%)/82 (75%)	0.264	26 (20%)/102 (80%)	11 (32%)/23 (68%)	0.106
Stroke/TIA *Yes/No*	1 (3%)/29 (97%)	12 (9%)/120 (91%)	0.264	1 (2%)/52 (98%)	12 (11%)/97 (89%)	0.037	9 (7%)/119 (93%)	4 (12%)/30 (88%)	0.278
aTHROMB *Yes/No*	1 (3%)/29 (97%)	23 (17%)/109 (83%)	0.036	3 (6%)/50 (94%)	21 (19%)/88 (81%)	0.016	13 (10%)/115 (90%)	11 (32%)/23 (68%)	0.003
aPLT *Yes/No*	1 (3%)/29 (97%)	24 (18%)/108 (82%)	0.030	4 (8%)/49 (92%)	21 (19%)/88 (81%)	0.040	20 (16%)/108 (84%)	5 (15%)/29 (85%)	0.568
PFO *Yes/No*	6 (20%)/24 (80%)	2 (2%)/130 (98%)	0.001	6 (11%)/47 (89%)	2 (2%)/107 (98%)	0.015	8 (6%)/120 (94%)	0 (0%)/34 (100%)	0.145
Coiling *Yes/No*			0.260			0.357			0.406
	0 (0%)/21 (70%)	6 (5%)/80 (61%)		1 (2%)/34 (64%)	5 (5%)/67 (61%)		4 (3%)/79 (56%)	2 (6%)/22 (65%)	
Kinking *Yes/No*	1 (3%)/20 (67%)	19 (14%)/68 (52%)	0.058	3 (6%)/33 (62%)	17 (16%)/55 (50%)	0.043	16 (13%)/68 (53%)	4 (12%)/20 (59%)	0.527
Tortuosity *Yes/No*	9 (30%)/12 (40%)	67 (51%)/20 (15%)	0.003	15 (28%)/21 (40%)	61 (56%)/11 (21%)	0.000	56 (44%)/28 (22%)	20 (59%)/4 (12%)	0.090
Arch type			0.543			0.529			0.687
***1***	11	63		22	52		56	18	
***2***	1	8		3	6		6	3	
***3***	0	1		0	1		1	0	
***6***	0	1		0	1		1	0	
Vasospasm *Yes/No*	1 (3%)/29 (97%)	3 (2%)/129 (98%)	0.563	1 (2%)/52 (98%)	3 (3%)/106 (97%)	0.604	4 (3%)/124 (97%)	0 (0%)/34 (100%)	0.386
ENT *Yes/No*	0 (0%)/30 (100%)	11 (8%)/121 (92%)	0.097	3 (6%)/50 (94%)	8 (7%)/101 (93%)	0.488	9 (7%)/119 (93%)	2 (6%)/32 (94%)	0.584
Dissection *Yes/No*	0 (0%)/30 (100%)	4 (3%)/128 (97%)	0.437	0 (0%)/53 (100%)	4 (4%)/105 (96%)	0.201	3 (2%)/125 (98%)	1 (3%)/33 (97%)	0.614
SAH *Yes/No*	2 (7%)/28 (93%)	8 (6%)/128 (94%)	0.585	2 (4%)/51 (96%)	8 (7%)/101 (93%)	0.307	4 (3%)/124 (97%)	6 (18%)/28 (82%)	0.006
ICH *Yes/No*	1 (3%)/28 93%)	1 (0.8%)/127 (96%)	0.336	2 (4%)/48 (90%)	0 (0%)/107 (98%)	0.100	2 (2%)/121 (95%)	0 (0%)/34 (100%)	0.613
3DCT			0.177			0.027			0.100
***HI1 ECASSII***	6	16		12	10		19	3	
***HI2 ECASSII***	5	26		9	22		25	6	
***PH1 ECASSII***	0	2		1	1		2	0	
***PH2 ECASSII***	1	0		1	0		1	0	
***Normal***	9	48		16	41		45	12	
Final mTICI			0.757			0.994			0.537
***0***	1	14		5	10		9	6	
***1***	2	7		3	6		7	2	
***2a***	3	11		5	9		11	3	
***2b***	11	37		17	31		41	7	
***2c***	3	16		6	13		15	4	
***3***	10	47		17	40		45	12	
Final aspiration mTICI			0.502			0.485			0.554
***0***	3	20		7	16		15	8	
***1***	3	14		4	13		13	4	
***2a***	1	16		3	14		14	3	
***2b***	11	32		18	25		37	6	
***2c***	2	11		5	8		10	3	
***3***	10	39		16	33		39	10	
Number of passes			0.124			0.192			0.828
***1***	16	56		26	46		57	15	
***2***	10	45		19	36		44	11	
***3***	4	23		7	20		21	6	
***4***	0	6		0	6		4	2	
***5***	0	2		1	1		2	0	
First pass success *Yes/No*	15 (50%)/15 (50%)	46 (35%)/83 (63%)	0.107	23 (43%)/29 (55%)	38 (35%)/69 (63%)	0.187	49 (38%)/76 (59%)	12 (35%)/22 (65%)	0.418
mTICI 2b-3 *Yes/No*	23 (77%)/7 (23%)	82 (62%)/50 (38%)	0.096	38 (72%)/15 (28%)	67 (61%)/42 (39%)	0.134	86 (67%)/42 (33%)	19 (56%)/15 (44%)	0.153
NIHSS initial	15.50	16.00	0.848	15.00	16.00	0.255	16.00	15.50	0.963
NIHSS admission	16.00	16.00	0.881	16.00	16.00	0.993	16.00	16.00	0.786
NIHSS ICU	11.50	14.00	0.294	13.00	14.00	0.234	13.50	16.00	0.249
NIHSS 24 h	6.00	12.00	0.028	8.00	12.00	0.063	10.00	14.00	0.205
NIHSS discharge	5.00	7.00	0.247	6.00	5.00	0.943	5.00	7.00	0.273
Exposure time	23.90	28.82	0.056	26.55	28.57	0.382	27.61	29.03	0.753
Absrobed dose	553.53	611.39	0.494	578.79	611.32	0.648	616.91	539.56	0.174
CM Volume	131.33	131.78	0.869	137.55	128.85	0.209	134.18	122.35	0.201
OTG	265.57	248.47	0.683	269.21	242.93	0.144	259.00	223.79	0.057
OTR	309.90	300.21	0.907	313.43	296.35	0.378	308.67	274.87	0.170
Extraccranial length ratio	1.11	1.27	0.000	1.18	1.27	0.000	1.22	1.31	0.004
Intracrianial length ratio	1.81	1.73	0.442	1.75	1.74	0.876	1.75	1.72	0.656
Overall length ratio	1.28	1.39	0.001	1.30	1.41	0.000	1.36	1.40	0.010
Occluded vessel diameter	2.93	2.85	0.887	2.92	2.84	0.987	2.95	2.56	0.033
Vessel to ID catheter ratio	1.79	1.75	0.940	1.79	1.74	0.963	1.81	1.55	0.015
Vessel to OD catheter ratio	1.51	1.47	0.882	1.51	1.47	0.984	1.53	1.30	0.013

mRS, modified Rankin Scale; rtPA, recombinant tissue plasminogen activator; AF, atrial fibrillation; BA, basilar artery; DB, diabetes; LIP, hypercholesterolemia; NT, hypertension; COR, coronary heart disease; TIA, transient ischemic stroke; aTHROMB, antithrombotic medication; aPLT, antiplatelet medication; PFO, patent foramen ovale; ENT, embolization in a new territory; SAH, subarachnoid hemorrhage; ICH, intracerebral hemorrhage; mTICI, modified Thrombolysis in Cerebral Infarction scale score; MCA-M1, middle cerebral artery M1 segment; MCA-M2, middle cerebral artery M2 segment; NIHSS, National Institute of Health Stroke Scale; CM, contrast medium (ml); OTG, onset-to-groin time (min); OTR, onset-to-recanalization time (min); ICA-T, internal carotid terminus; ICU, intensive care unit; ID, inner diameter; OD, outer diameter; 3DCT, flat-panel computed tomography; HI1, hemorrhagic infarction type 1; HI2, hemorrhagic infarction type 2; PCA-P2, posterior cerebral artery P2 segment; PH1, parenchymal hematoma type 1; PH2, parenchymal hematoma type 2.

### Spearman's rank correlation analysis

Age was significantly correlated with several clinical characteristics, including the mRS status at 12 months after stroke (*R* = 0.464, *p* = 0.000); moderately with the pre-stroke mRS status (*R* = 0.320, *p* = 0.000), death at 12 months after stroke (*R* = 0.327, *p* = 0.003), and atrial fibrillation (AF) (*R* = 0.306, *p* = 0.000); and weakly with hypertension (*R* = 0.275, *p* = 0.000), treatment with antithrombotic agents (*R* = 0.285, *p* = 0.000), and the presence of PFO (*R* = −0.253, *p* = 0.0001). Significant correlations were found in terms of anatomical characteristics, namely, a strong correlation with the presence of carotid tortuosity (*R* = 0.408, *p* = 0.000), extracranial length ratio (*R* = 0.487, *p* = 0.000), and overall length ratio (*R* = 0.467, *p* = 0.000). No significant associations were found with coiling, kinking, or intracranial length ratio.

Modified Thrombolysis in Cerebral Infarction (mTICI2b-3) scale score showed uniform inverse correlations with several clinical, anatomical, and procedure-related parameters, namely, NIHSS in the intensive care unit (ICU), after 24 h, and at discharge (*R* = −0.212, *p* = 0.014, *R* = −0.266, *p* = 0.002, and *R* = −0.231, *p* = 0.011, respectively); mRS at 90 days (*R* = −0.361, *p* = 0.000), 12 months, and death at 12 months (*R* = −0.434, *p* = 0.001; and *R* = −0.272, *p* = 0.013, respectively); the extracranial and overall length ratios (*R* = −0.210, *p* = 0.035, and *R* = −0.258, *p* = 0.009, respectively); exposure time (*R* = −0.481, *p* = 0.000); absorbed dose (*R* = −0.434, *p* = 0.000); contrast medium volume (*R* = −0.226, *p* = 0.004); the presence of any complications (*R* = −0.234, *p* = 0.003); the use of a stent-retriever (*R* = −0.647, *p* = 0.000); and the number of passes (*R* = −0.246, *p* = 0.002).

Successful recanalization was also significantly but weakly correlated with tortuosity (R = −0.246, *p* = 0.01). No significant association was observed with first-pass success. Kinking and coiling did not show any significant relationship with the parameters for successful recanalization.

The presence of coiling was weakly but significantly associated with embolization in new territories during ADAPT (*R* = 0.264, *p* = 0.006); however, it showed no significant association with other periprocedural complications, such as vasospasm, dissection, and subarachnoid or intracerebral hemorrhage. None of these adverse events were associated with kinking or tortuosity. First-pass success was not significantly correlated with vessel tortuosity (*p* = 0.075).

Atrial fibrillation was inversely correlated with tandem lesion (*R* = −0.237, *p* = 0.003), first-pass success (*R* = −0.156, *p* = 0.01), and onset-to-groin/reperfusion times (*R* = −0.158, *p* = 0.04; and *R* = −0.163, *p* = 0.05, respectively). Its presence was also associated with a higher mRS status after 90 days (*R* = 0.256, *p* = 0.01).

### Successful aspiration

**Figure 2:**
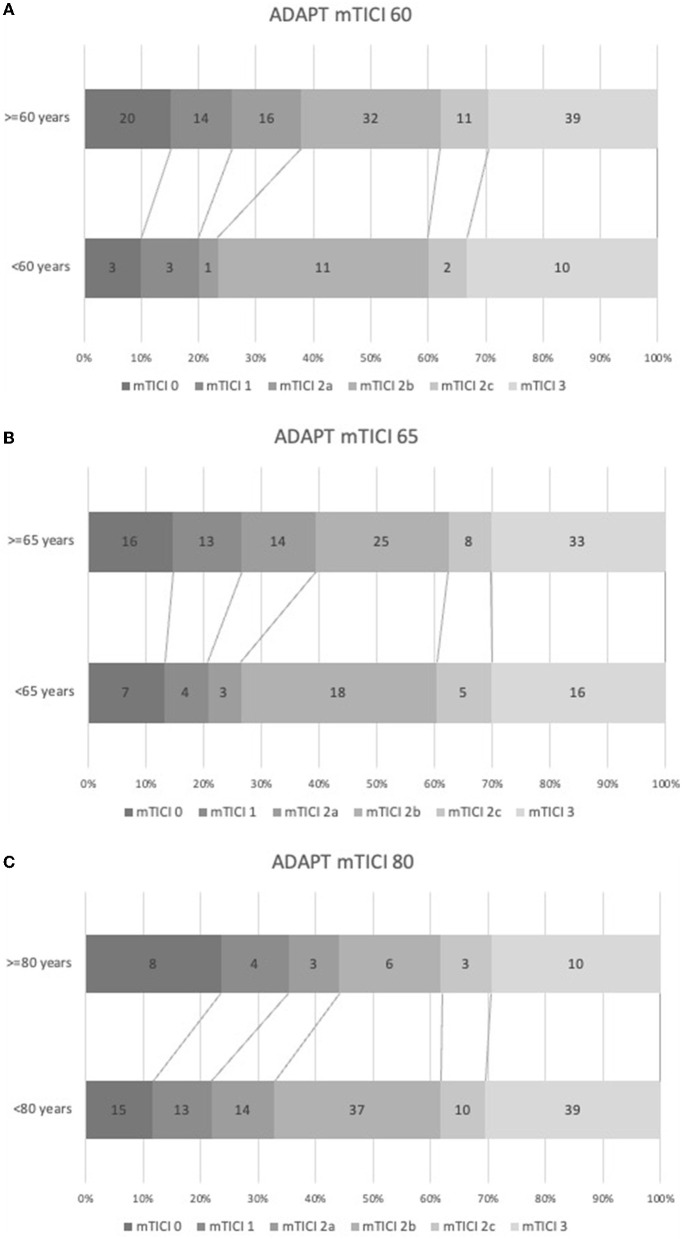
Stacked bar charts showing performance of ADAPT in selected age groups, none of the differences were statistically significant.

Although successful aspiration-based recanalization rates decreased with increasing age, the differences between the subgroups were not statistically significant. In the subgroup aged < 60 years (23 of 30 patients), mTICI2b-3 was achieved in 77% of the cases. In the subgroup aged ≥ 60 years (82 of 132 patients), mTICI2b-3 was achieved in 62% of the cases. When the division was made at 65 years, mTICI2b-3 in those aged < 65 years (38 of 53 patients) fell to 72%, while that in those aged ≥ 65 years (67 of 109 patients) fell to 61%. The aspiration recanalization rate in patients aged < 80 years (86 of 128 patients) was 67%, while it dropped to 56% in those aged ≥ 80 years (19 of 34 patients). A comparison between the extreme subgroups, that is, < 60 years vs. ≥ 80 years, did not yield a statistically significant change (*p* = 0.068). The percentages of successful recanalization and first-pass success, depending on the occlusion site, are shown in Table 2. Stacked bar charts comparing the aspiration results in different age subgroups are shown in Figure 2.

**Table 2:** Percentage of successful recanalization and first-pass success (FPS) depending on occlusion site and age group.

		**Overall**	** < 60**	**≥60**	** < 65**	**≥65**	** < 80**	**≥80**
Carotid-T	mTICI2b-3	63.5% *(33/52)*	83.3% *(10/12)*	57.5% *23/40*	75.0% *15/20*	56.3% *18/32*	65.9% *29/44*	44.4% *4/9*
	FPS	30.8% *16/52*	41.7% *5/12*	27.5% *11/40*	35.0% *7/20*	28.1% *9/32*	31.8% *14/44*	22.2% *2/9*
MCA-M1	mTICI2b-3	63.6% *49/77*	71.4% *10/14*	61.9% *39/63*	61.8% *34/55*	68.2% *15/22*	65.0% *39/60*	58.8% *10/17*
	FPS	40.3% *31/77*	64.3% *9/14*	34.9% *22/63*	38.2% *21/55*	45.5% *10/22*	41.7% *25/60*	35.3% *6/17*
MCA-M2	mTICI2b-3	69.6% *16/23*	100.0% *2/2*	66.7% *14/21*	66.7% *4/6*	70.6% *12/17*	75.0% *12/16*	57.1% *4/7*
	FPS	39.1% *9/23*	0.0% *0/2*	42.9% *9/21*	33.3% *2/6*	41.2% *7/17*	43.8% *7/16*	28.6% *2/7*

ex. 33/52 means 32 patients had mTICI2-3 result out of 52 patients with carotid-t occlusion and so on.

### Effect of vessel tortuosity

Several correlations associated with vessel tortuosity have been described previously.

Overall, the presence of tortuosity led to a significant increase in the exposure time (*p* = 0.011), followed by the absorbed dose (*p* = 0.003). Notably, the opposite trend was observed for the contrast medium volume (*p* = 0.728). The time from onset-to-groin access and the time from onset-to-recanalization differed significantly between patients with intracranial and extracranial tortuosity (*p* = 0.004 and *p* = 0.003; *p* = 0.000 and *p* = 0.004, respectively).

Neither intracranial nor extracranial tortuosity was significantly associated with reperfusion-related complications in either subgroup (i.e., vasospasm, embolization in a new territory, dissection, and intracerebral hemorrhage), apart from SAH in patients aged ≥80 years (*p* = 0.04) with extracranial vessel elongation. The presence of intracerebral tortuosity was associated with a significant increase in the incidence of SAH with increasing age [≥ 60 years (*p* = 0.040), ≥65 years (*p* = 0.033), and ≥ 80 (*p* = 0.021)].

Extracranial tortuosity was observed significantly more frequently in patients aged ≥80 years with hypercholesterolemia and hypertension (*p* < 0.05). Analogous observations were made in the case of intracerebral tortuosity only for hypercholesterolemia (*p* < 0.05).

History of stroke or transient ischemic stroke and AF; current antithrombotic or antiplatelet therapy; and diagnosis of diabetes did not differ significantly between patients with tortuous intracerebral or extracerebral vessels.

The patient status (i.e., NIHSS, mRS, and death) did not differ significantly with the presence of extracranial tortuosity, regardless of the time of assessment (24 h, 30 days, 3 months, and 12 months after treatment) for any of the subgroups. Overall, NIHSS after 24 h and at discharge were significantly different in patients with intracerebral tortuosity (*p* = 0.023 and *p* = 0.050, respectively).

The ROC analysis did not show any predictive cutoff values for the ratios of inner/outer catheter to the occluded vessel diameters with regard to successful recanalization or first-pass success in any of the groups.

## Discussion

Regardless of the mixed results reported by previous studies on aspiration-based thrombectomy, randomized multicenter trials and meta-analyses have demonstrated that ADAPT is successful in achieving recanalization of large occluded intracranial vessels and is non-inferior to stent-retriever-based thrombectomy (5, 18–20). The data showed no significant differences in terms of the success of angiography or clinical outcomes. Successful recanalization determines the prognosis (21). It is believed to be associated with a younger age, a shorter onset-to-clot contact time, an isolated middle cerebral artery M1 segment occlusion, and an rtPA administration (22–26).

Age was previously reported to be associated with clinical outcomes after mechanical thrombectomy, with poorer mRS scores associated with increasing age (27, 28). The older the patient, the higher the incidence of comorbidities, thereby impeding the ability to recover from a stroke. Atrial fibrillation rate increases with age and is associated with a 5-fold increase in the risk of stroke. In a recent meta-analysis, successful reperfusion rates (mTICI-2b/3) were insignificantly different in patients with and without AF (OR, 1.11 [95% CI, 0.78–1.58]; *p* = 0.57). Similarly, symptomatic intracerebral hemorrhage rates were comparable (OR, 1.05 [95% CI, 0.84–1.31]; *p* = 0.68). However, it was not reflected in mortality and functional independence after mechanical thrombectomy. Patients suffering from AF tend to present a lower Alberta Stroke Program Early CT Score (ASPECTS) and a higher NIHSS (29, 30).

In our study population, the mRS at discharge did not differ significantly, regardless of age. Diverging prognosis was observed with increasing observation time, that is, 30 days post-stroke, and the clinical status differed significantly only between younger patients and those aged ≥80 years (*p* = 0.046). The difference was more significant in these subgroups after 90 days (*p* = 0.011). All subdivisions differed significantly after 12 months. The effect of comorbidities on clinical outcomes could explain why the neurological status measured by NIHSS did not differ significantly between the subgroups, regardless of the timeframe (initial, on admission to the hospital, in the ICU, after 24 h, or at discharge).

The presence of AF could exclude the administration of rtPA, in the case of anticoagulant treatment (30). There are mixed reports on the benefits of intravenous thrombolysis (IVT) prior to interventional stroke treatment. The *post hoc* analysis of a recent randomized trial published by Rinkel et al. reported that patients treated with ADAPT had poorer clinical outcomes without IVT; however, it had no impact on successful reperfusion (31). In our series, rtPA showed a weak positive correlation with successful reperfusion (*R* =0.2, *p* = 0.014) and was not associated with SAH or intracerebral hemorrhage.

The MR CLEAN Registry showed that the reperfusion rates favored aspiration compared with stent-retrievers in anterior circulation (ICA-T – OR 1.3, MCA-M1 – OR 1.3, and MCA-M2 – OR 1.2) (32). The reported successful reperfusion percentages were 73% for ICA-T, 72% for MCA-M1, and 72% for MCA-M2. Although these results were comparable with our results (64, 64, and 70%, respectively), the first-pass success rates were inferior (43, 58, and 53% vs. 31, 40, and 39%, respectively). This observation could be partially explained by the inclusion of more diverse age groups, which included both younger and older patients (range 35–94 years in our group vs. 62–80 years), where we observed better reperfusion rates in those aged < 60 years.

Advanced age is one of the several factors associated with carotid tortuosity, which also includes hypertension, elevated body mass index, and atherosclerosis (8, 33, 34). Their relative capacity to alter the length of the arteries is disputed. Nevertheless, everyday neurovascular interventions, *inter alia*, and stroke treatment are affected by carotid anatomy. Benson et al. found that tortuosity was present in ~40% of patients undergoing stroke treatment (35). In our study, tortuosity alone was present in up to 77% of patients aged ≥60 years and 83% of those aged > 80 years. Kinking, which becomes less prevalent with age, was present in 23% of patients, whereas coiling affected a maximum of 8% of those patients aged >80 years). No coiling was observed in patients aged < 60 years.

Kinks were reported to negatively affect the rates of successful recanalization before; however, no significant complications occurred during mechanical thrombectomy (35). In our study, tortuosity was significantly but weakly correlated with the success of angiography (*R* = – 0.246, *p* = 0.01); however, no association was found with any procedure-related adverse events. This could be explained by different anatomy assessment methodologies being used and different age groups being evaluated. We divided the carotid anatomy into two segments: intracranial and extracranial. Only intracranial segment tortuosity was significantly correlated with SAH when performing ADAPT, regardless of age.

Tortuous vessels present a challenge in the delivery of aspiration catheters. Theoretically, the efficacy of ADAPT could be improved by using larger aspiration catheters, a notion that has already been supported by *in vitro* and animal experiments (36). The larger the bore of the catheter, the more difficult the delivery due to hindrance by the meandering anatomy and branching arteries. Successful aspiration relies on adequate catheter diameter. Kyselyova et al. found that the vessel diameter and the catheter-to-vessel ratio affected the effectiveness of clot aspiration (37). In their study, the higher the ratio, the higher the rate of the first aspiration success.

In the COMPASS trial, ADAPT was performed using a 0.068-inch catheter, regardless of the occlusion site. We did not evaluate the performance of specific catheters since their deliverability was not assessed in this study. The catheter characteristics were tapered to the inner and outer diameters, especially in relation to the closed vessel diameter. We performed the ROC analysis to calculate the predictive cutoff value for the ratios of the inner/outer catheter to the occluded vessel diameters with regard to successful recanalization or first-pass success but of no avail. We found that a larger ratio was significantly associated with SAH after ADAPT.

Our study has some limitations. These limitations included its retrospective study design and relatively small sample size. Moreover, the number of passes and the choice of catheters were at the discretion of the operator. Our department's policy permits three unsuccessful attempts before changing the device. We did not consider any attempts where the aspiration catheter could not be delivered to the thrombus in this study. Finally, several calculations, especially with carotid terminus or tandem occlusions, were based on the contralateral artery measurements.

In unselected patients, the successful aspiration-based recanalization rate decreased with increasing age; however, this difference was not significant. Clinical outcomes, such as NIHSS, mRS, and death, did not differ significantly with regard to carotid tortuosity, regardless of the time of assessment (24 h, 30 days, 3 months, and 12 months). Neither intracranial nor extracranial tortuosity was significantly associated with reperfusion-related complications in the subgroups. SAH was significantly more prevalent in the oldest subgroup, although it was clinically inconsequential. In addition, larger catheter-to-occluded vessel ratios were associated with SAH.

## Data availability statement

The raw data supporting the conclusions of this article will be made available by the authors, without undue reservation.

## Ethics statement

The studies involving human participants were reviewed and approved by Institutional Review Board, Military Institute of Medicine. The patients/participants provided their written informed consent to participate in this study.

## Author contributions

The authors confirm contribution to the paper as follows: study conception and design: JN. Data collection: JN, AP, PZ, AD, and MW. Analysis and interpretation of results: JN and AP. Draft manuscript preparation: JN and PP. Manuscript revision: MW, PP, and JS. Supervision: PP and JS. All authors reviewed the results and approved the final version of the manuscript.
